# Advances in the research of nano delivery systems in ischemic stroke

**DOI:** 10.3389/fbioe.2022.984424

**Published:** 2022-10-21

**Authors:** Yi-Xuan Li, Hong-Bo Wang, Jian-Bo Jin, Chun-Lin Yang, Jing-Bo Hu, Jing Li

**Affiliations:** ^1^ Faculty of Materials Science and Chemical Engineering, Ningbo University, Ningbo, China; ^2^ Department of Pharmacy, Ningbo University Affiliated Yangming Hospital, Yuyao, China

**Keywords:** ischemic stroke, blood-brain barrier, nano delivery system, polymer, endogenous vesicles

## Abstract

Ischemic stroke is the most common type of cerebrovascular disease with high disability rate and mortality. The blood-brain barrier (BBB) protects the homeostasis of the brain’s microenvironment and impedes the penetration of 98% of drugs. Therefore, effective treatment requires the better drug transport across membranes and increased drug distribution. Nanoparticles are a good choice for drugs to cross BBB. The main pathways of nano delivery systems through BBB include passive diffusion, adsorption-mediated endocytosis, receptor-mediated transport, carrier-mediated transport, etc. At present, the materials used in brain-targeted delivery can be divided into natural polymer, synthetic polymers, inorganic materials and phospholipid. In this review, we first introduced several ways of nano delivery systems crossing the BBB, and then summarized their applications in ischemic stroke. Based on their potential and challenges in the treatment of ischemic stroke, new ideas and prospects are proposed for designing feasible and effective nano delivery systems.

## Introduction

Ischemic stroke, also known as cerebral infarction, is the most common type of cerebrovascular disease. It accounts for about 70% of all acute cerebrovascular diseases, mainly in the middle-aged and elderly groups. Ischemic stroke is a general term for necrosis of brain tissue caused by insufficient blood supply to the brain due to stenosis or occlusion of the arteries supplying blood to the brain (carotid and vertebral arteries) (Herpich and Rincon, 2020). According to the etiology, it can be divided into atherosclerosis, cardiogenic embolism, arteriolar occlusion, other definite etiology and unknown etiology. According to the location of infarction, it can be divided into total anterior circulation infarction, partial anterior circulation infarction, posterior circulation infarction and lacunar infarction (Jolugbo and Ariëns, 2021). In addition, there is a special type of cerebral infarction, perinatal neonatal stroke, which is mainly manifested as focal nerve damage in the early stage of brain development, including neonatal arterial ischemic stroke, cerebral sinus venous thrombosis and neonatal hemorrhagic stroke (Mineyko et al., 2020). Ischemic stroke has no specific early symptoms, which vary according to the infarct site, size, blocked blood vessels and other reasons. Cerebral infarction is one of the largest causes of death in China at present. The mortality in the acute phase is up to 5%–15%, and the disability rate in the surviving patients is even as high as 50%. It can lead to paralysis, aphasia, blindness, etc., which will bring a heavy psychological burden to the patients, their families and the society (Paul and Candelario-Jalil, 2021). While suffering from ischemic cerebral infarction, it will also be accompanied by a variety of complications, including limb paralysis and movement disorders, speech and swallowing difficulties, memory loss and thinking difficulties, emotional disorders, headache and decreased self-care ability. Although there is no way to cure ischemic stroke, it can slow down the progress of disease. Therefore, it is most important to find an effective treatment (Ajoolabady et al., 2021).

Nano delivery systems are typically between 10 and 1,000 nm in size. They are usually composed of natural polymers or a list of synthetic polymers materials, and can be used as carriers for delivering drugs (Wong et al., 2012). Nano delivery system is a kind of sub-particle drug carrier delivery system, which belongs to nanometer level microscopic category. It can adjust the release rate of drug, increase the permeability of biofilm, improve the distribution in the body, and increase the bioavailability, etc. (Li et al., 2021a). Overall, nano delivery systems have unique advantages in many ways. The small particle size and large specific surface area of nanoparticles can embed hydrophobic substances and improve the hydrophilic of drugs. Mediated by the targeted group, the drug delivery system delivers the drug to lesion, reducing side effects. The release of the encapsulated drugs can be prolonged, which is beneficial to increase action time, and reduce the frequency of administration (Tian et al., 2021a).

Ischemic stroke is characterized by insufficient cerebral blood supply and large area necrosis of nerve cells due to occlusion of local cerebral artery. The focal areas are divided into core infarct zone and ischemic penumbra (Liu et al., 2018). There is a large amount of cell death in the core infarct area, and blood flow is less than 20% of normal blood flow. Ischemic penumbra blood flow is 25–50 percent of normal blood flow, and nerve cells are endangered but still treatable (Wang et al., 2021a). If left untreated, the core infarcts can spread outwards and worsen the disease. The effective drug transport across the BBB and good brain distribution are crucial during treatment (Pardridge, 2012). The BBB is a barrier between blood and brain tissue formed by capillary endothelial cells. It is composed of closely arranged monolayer endothelial cells, surrounded by basement membrane, pericytes and glial cells, and has a sugar calyx composed of carbohydrates on the inner surface (Xie et al., 2019). As a unique physiological barrier of the brain, BBB not only protects the steady-state balance of the brain’s own microenvironment, but also hinders the penetration of 98% of drugs (Umlauf and Shusta, 2019). Therefore, whether it can penetrate BBB and the percentage of penetration become one of the evaluation directions of drugs for the treatment of brain injury.

**FIGURE 1:**
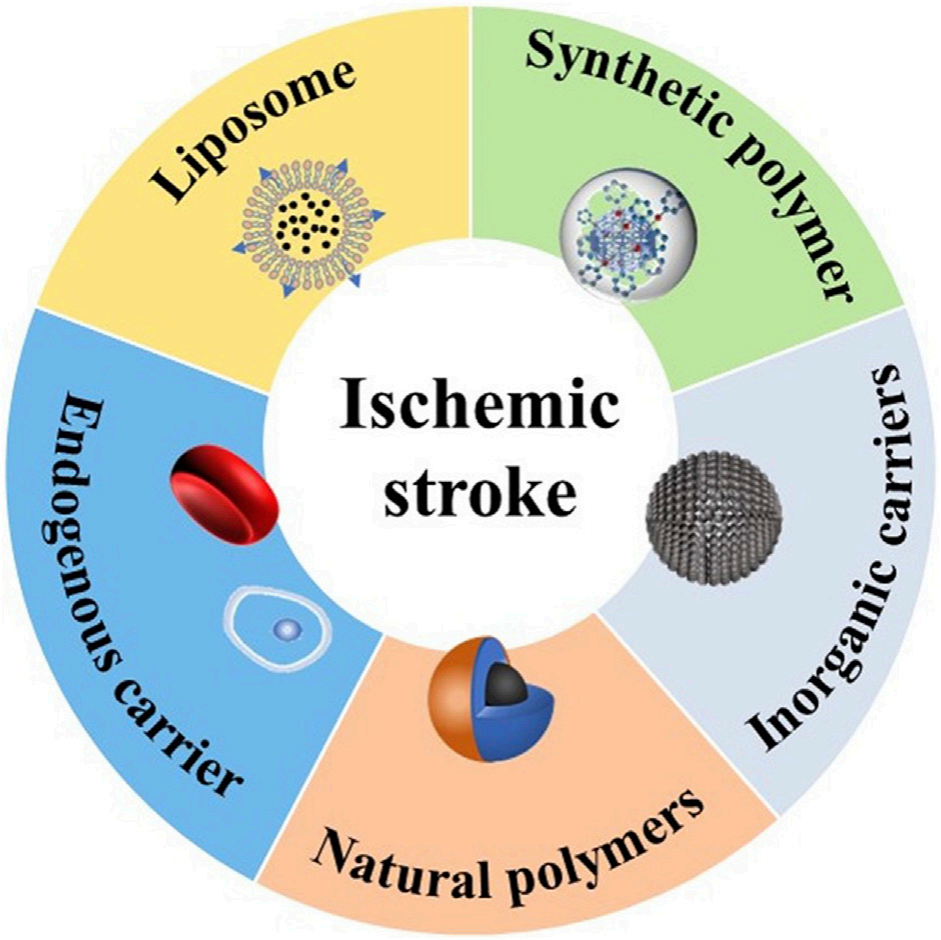
Treatment of ischemic stroke with nanoparticles.

In recent years, great progress has been made in the delivery of brain-targeted nano delivery systems through BBB, which provides a promising treatment of ischemic stroke (Dong et al., 2020). In this review, we first introduce the methods of drug crossing BBB, and then summarize the nano delivery system for treatment of acute ischemic stroke based on the difference of particle materials (Figure 1, Table 1).

**TABLE 1:** List of nanomaterials for ischemic stroke therapy.

Materials	Drug	Strategies	Major outcomes	References
mPEG-b-P (DPA-co-HEMA)-Ce6	Rapamycin	pH-sensitive	Drug concentration in ischemic sites, neuroprotective effects	Cheng et al. (2021)
PEG, PCL, enzyme-cleavable peptides	Glyburide	Protease-responsive	High efficiency in penetrating ischemic brain area, safe material and low toxicity	Guo et al. (2018)
PG2HR	pDNA	HO-1 gene	Reduce the apoptosis level of ischemic brain tissue, reduce infarct area, low cytotoxicity and high gene transfer efficiency	Lee et al. (2021)
PEG-b-PMNT	t-PA	ROS scavenging	Reduce cerebral infarction volume after cerebral ischemia and improve neurological function defect	Mei et al. (2019)
Dextran	NR2B9C	ROS/pH sensitive release	Prolong the systemic circulation of NR2B9C, enhance the active targeting effect of NR2B9C on ischemic area, and alleviate ischemic brain injury	Lv et al. (2018)
Hydroxyethyl starch	SAG	pH-sensitive release	Significantly promote angiogenesis and reduce vascular permeability	Yang et al. (2021a)
Hydrogel polysaccharide	The gold standard fibrinolytic–alteplase	P-selectin-targeting	Enhance the thrombolytic activity of the clinical drug *in vivo*, reduce cerebral infarction lesions after ischemia and blood brain barrier permeability	Zenych et al. (2021)
EVs		Anti-inflammatory	Target the lesion region of the ischemic brain and suppress poststroke inflammation	Tian et al. (2021b)
Platelet membrane	rt-PA	Thrombus-targeting	Lower risk of bleeding complications, effective aggregation at the thrombi site, and significantly enhance thrombolytic activity	Xu et al. (2020)
PLGA	Rapamycin	Inflammatory-targeting	Rmproved area of injury, greatly improve neurological score and infarct volume	Wang et al. (2021b)
SPIO-platelet	Piceatannol	Thrombus-targeting	Reduce neutrophil infiltration, reduce infarct size, and monitor inflammatory neutrophil in real time	Tang et al. (2019)
Dextran-platelet membrane	rtPA/ZL006e	Thrombus-targeting	Significantly enhance the efficacy of ischemic stroke, decrease the level of ischemic area and reactive oxygen species	Xu et al. (2019)
Neutrophil membrane	Resolvin D2	Inflammatory brain endothelium- targeting	Reduce the inflammatory response of ischemic stroke, improve the neurological function of mice	Dong et al. (2019)
MnO_2_	Fingolimod	PH-sensitive release	Reduce oxidative stress, promote the transformation of M1-type microglia to M2-type microglia, enhance the survival of damaged neurons	Li et al. (2021b)
SPIO	siRNA	SiRNA delivery and imaging	Infarct volume was reduced, functional defect and partial anisotropy (FA) values were increased, and fiber count was increased	Wang et al. (2019)
CeO_2_	Edaravone	ROS-scavenging	Enhance intracerebral uptake while effectively protecting the blood-brain barrier, greatly reduce harmful side effects and sequelae	Bao et al. (2018)
CeO_2_	ZIF-8	Catalytic and antioxidative activities	Prolong blood circulation time, reduce clearance rate, improve the penetration ability of blood-brain barrier, effectively inhibit lipid peroxidation, and reduce neuronal oxidative damage and apoptosis in brain tissue	He et al. (2020)
γ-Fe_2_O_3_	L-arginine	Lesions-targeting	Rapid targeting, *in situ* production of NO, vascular dilation, blood flow recovery	Li et al. (2020)
IONP	MAC	Lesions-targeting	Promote the anti-inflammatory response, angiogenesis and anti-apoptosis of ischemic brain injury, reduce the volume of cerebral infarction, improve motor function	Kim et al. (2020)
Liposome	T7-SHp	Lesions-targeting	Improve the infarct volume, nerve function defect and histopathological degree	Zhao et al. (2016)
BA	AMD3100	Targeted-AMD3100, pH-sensitive release	Target delivery to ischemic brain tissue with high efficiency, accelerate drug preferentially release to ischemic brain tissue	Zhang et al. (2022)

## Blood-brain barrier

BBB is composed mainly of non-wall-less endothelium, which is characterized by the presence of tight junctions, forming a cell barrier that is almost impossible to penetrate (Abbott et al., 2010). Among them, the brain capillary endothelium (BMECs) has obvious structural features compared with the capillary in other organs (Kutuzov et al., 2018). First, the lack of pores in the capillary and the overlapping coverage and tight junctions between the cells greatly reduce the passage of drugs between the cells. Second, the endothelial cells are surrounded by a continuous layer of basement membrane. Third, there are many perivascular feet of astrocytes outside the basement membrane. All of these structures form the capillary membrane of the brain, which forms the protective BBB of the brain tissue. At present, the main pathways through BBB include passive diffusion, adsorption-mediated transport, receptor-mediated transport, carrier-mediated transport, and cell-mediated transport (Chen and Liu, 2012; Patel and Patel, 2017; Kim et al., 2019).

### Passive diffusion

Passive diffusion is a kind of membrane transport of ions and small molecules without any special transport medium and carrier under the condition of concentration difference and potential difference (Cocucci et al., 2017). The diffusion of matter into the brain is divided into paracellular diffusion and anti-cellular diffusion. Based on the special structure of BBB, passive diffusion is very limited. Therefore, only non-dissociative, lipophilic and low molecular weight molecules can freely diffuse through the endothelial membrane, thus passively crossing BBB, the rest of the molecules need to enter through other ways (Li et al., 2017). Although passive diffusion is not the main way for drugs to penetrate BBB, it is likely to increase the transport speed and percentage of drugs as an auxiliary process. Kiyohiko et al. investigated the assisted processes of passive diffusion and carrier transport in drug transport (Sugano et al., 2010). Drug absorption experiments in intestinal epithelial cells were used by them to demonstrate that passive transport and vector-mediated transport coexist. Moreover, passive permeation is an important (and often major) factor in drug-like membrane permeation (Sugano et al., 2010). Although this study was not aimed at brain endothelial cells, it has reference value for future research.

### Adsorption mediated transport

According to different mechanisms, cell endocytosis can be divided into receptor-mediated transport (RMT) and adsorption mediated transport (AMT). AMT is a transport pathway caused by the electrostatic interaction between polycations and the negative components of the plasma membrane in the lumen. For example, interaction with acidic residue anion sites of acidic glycoproteins (Scherrmann, 2002). AMT is one of the main ways for drugs to enter the central nervous system through BBB from the systemic circulation, helping to build more brain targeted drug delivery systems mediated by adsorption. The duration of AMT is short and nonspecific. Therefore, the combination of drugs and cationic groups to construct nano drug delivery particles can improve the chances of drugs passing through BBB (Chen and Liu, 2012). In addition to the construction of nano delivery systems, it is important to investigate the endocytosis of BBB. By focusing on major facilitator superfamily domain containing 2a (MFSD2a), Andreone et al. revealed how central nervous system (CNS) endothelial cells ensure normal BBB function by inhibiting cellular metabolism (Andreone et al., 2017). What’s more, they confirmed that the lipid composition of CNS endothelial cells played an important role in the regulation of cell turnover and barrier permeability (Andreone et al., 2017). Although AMT provides a good strategy of drug delivery, the efficiency of drug delivery still needs to be improved.

### Receptor mediated transport

RMT is a way for extracellular macromolecules to selectively bind with receptors and then enter cells (Broadwell, 1989). It is also one of the most promising non-invasive methods to overcome the BBB (Urayama et al., 2008). The specific process of RMT is as followed: the macromolecule binds to the receptor protein on the cytoplasmic membrane, and the cell membrane is sunken to form a lipid vesicle containing the macromolecule (also known as endocytic vesicle). The vesicles appearing in the cells fuse with the intracellular body, and then fuse with the lysosome. The endocytic material is finally degraded internally (D et al., 2020). Unlike AMT, the RMT process has high selectivity, which is important for targeted drug delivery. There are many receptors available in brain endothelial cells, such as apolipoprotein receptor, transferrin receptor, sialic acid receptor, mannitol receptor and so on. The transferrin receptor (TFR), expressed on the endothelium of the brain, is a good target action site and can also reduce the distribution of drugs in non-target sites (Huang et al., 2021). Lactoferrin, a member of the transferrin family, is covalently coupled to PEG coated Fe_3_O_4_ nanoparticles and acts on the lactoferrin (LF) receptor in brain endothelial cells (Qiao et al., 2012). They successfully demonstrated the delivery of LF-receptor-mediated endocytic nanoparticles across the BBB (Qiao et al., 2012). Moreover, the use of lipid bilayer liposomal transferrin colloids was another strategy for obtaining highly effective drug delivery to the brain (Johnsen and Moos, 2016). The low-density lipoprotein receptor (LDLR) is highly expressed in the endothelial cells of the brain. The incorporation of PEG-PHDCA nanoparticles into cells was confirmed by observing the intracellular movement of nanoparticles in cell fractionation and confocal microscopy (Kim et al., 2007). Then, the LDLR block experiment showed that LDLR-mediated endocytosis was involved in the transport of nanoparticles (Kim et al., 2007).

In addition, RMT is the main mode of transport for effective antibody therapies in nervous system. The expresses endogenous expressed on BBB proteins that transport therapeutic drugs to the CNS. Steffen et al. examined the uptake of antibodies to several potential protein targets in the brain (Zuchero et al., 2016). Because receptors are not static in the body, there are a number of factors that need to be considered when delivering drugs *via* RMT, which are closely associated with the expression of the receptor in pathological conditions, the receptor before and after treatment, and age-dependent RMT transport efficiency (Yang et al., 2020).

### Other transport routes

Carrier-mediated transport (CMT), also known as alienation diffusion, is by means of a special carrier within the membrane transport way. CMT does not require energy, but with saturation and high specificity (Tsuji and Tamai, 1999). Common particle transportation systems include hexose transportation system, amino acid transportation system, monocarboxylic acid transportation system, amine transportation system, etc. (Han and Jiang, 2021). The combination of nanotechnology and CMT may promote the penetration of BBB, realize the brain targeted drug delivery and improve delivery efficiency. There have been many reports on CMT before, but most of them were associated with the cell-mediated immune response and its harm to the body (Klotz et al., 2016). Cell-mediated drug delivery nanoparticles have become one of the main research objects (Burrack et al., 2018). Maxime et al. decorated poly (ethylene glycol)-modified polystyrene nanoparticles into CD4^+^ T_EM_ cells by using thiol–maleimide coupling chemistry (Ayer et al., 2021). The results of 3D reconstructions of nine cells showed that 105 nanoparticles were fixed to the cells, which demonstrated that the activation effect/memory CD4^+^ T_EM_ cells could be used as a carrier for the delivery of polymer nanoparticles across the BBB (Ayer et al., 2021). Many cells have the ability to cross the BBB themselves, which allows the drugs to be delivered to the ischemic site without injury, increasing distribution in the brain. Therefore, cell-mediated drug delivery nanoparticles have great potential in the treatment of ischemic stroke.

## Application of nano delivery systems in ischemic stroke

Nano delivery systems have been widely used in various diseases. The rapid development of nanotechnology also provides a good opportunity for the treatment of ischemic stroke (Kim et al., 2018). The modification on nanoparticles potentially increases the probability and concentration of drugs crossing the BBB into the ischemic site, which has led to the development of a variety of biomaterials as drug carriers. (D'Souza et al., 2021). At present, nanocarriers can be divided into natural polymer, synthetic polymer, liposome, inorganic materials and endogenous vesicles.

### Nanoparticles

#### Synthetic polymer

The synthetic polymers used for carriers mainly include polylactic acid, Poly (lactic acid-glycolic acid) copolymer (PLGA), polyacrylate, polycaprolactone, etc. Synthetic polymer are characterized by the good purity, safety, low cost and repeatability (D’Souza et al., 2021).

PLGA is a kind of biodegradable functional polymer, which is randomly polymerized by two monomers (lactic acid and glycolic acid). It has good biocompatibility, non-toxicity, good performance of forming capsules and films, and is widely used for pharmaceutical and medical engineering materials. Han et al. proposed an innovative nanotechnology-based autocatalytic targeting approach (Han et al., 2016). PLGA was used to parcel the BBB regulator lexiscan (LEX) and NEP1-40 (Nogo-66 receptor antagonist) to form nanoparticles (PLGA-CTX/LEX NPs), which improved the drug permeability across BBB and brain distribution. PLGA-CTX/LEX NPs crossed the BBB through RMT or AMT *via* ligand surface coupling and positive charge display (Han et al., 2016). This strategy combined traditional targeted drug delivery with a new secondary autocatalytic mechanism to significantly reduce infarct size, and was suitable for systemic delivery of ischemic stroke. The PLGA-based nanoparticles can also harness abnormal levels of microRNA for stroke by binding different anti-Mir-oligonucleotides. The nanoparticles containing NA and Ps-based anti-mirS-141-3p were used to evaluate the therapeutic effect in post-stroke mice. After treatment, mir-141-3p levels in brain tissue and infarct injury were significantly reduced. PNA and Ps-based anti-mirS-141-3p loaded nanoparticles probe for the treatment of ischemic stroke were successfully established (Dhuri et al., 2021). Since the main mechanism of neuronal death following ischemia-reperfusion injury is reactive oxygen species (ROS) as well as the resulting apoptotic cascade, the outer mitochondrial membrane protein mitoNEET is an excellent therapeutic target (Saralkar et al., 2020). After binding to PLGA, the hydrophobic drug NL-1 (a specific ligand of mitoNEET) can cross the BBB *via* the caveolar-mediated endocytosis pathway in an energy-dependent manner, effectively decreasing the production of hydrogen peroxide after cellular ischemia and reducing apoptosis (Saralkar et al., 2020). Plasminogen activator (PA) is used for thrombosis, but is rarely recommended alone because of rapid drug metabolism and the bleeding risk (Zenych et al., 2020). Marianne et al. formed nano-CAT SOD-PLGA NPs by encapsulating antioxidant Catalase (CTA) and Superoxide dismutase (SOD) in PLGA (Petro et al., 2016). In the rat model of carotid artery injection of recombinant human tissue-type plasminogen activator (tPA), the drug was administered continuously. The results showed that PLGA-NPs could protect the BBB from ROS damage, reduce inflammatory reaction, protect neuronal cells from apoptosis and inhibit the formation of edema (Petro et al., 2016). In addition to vector delivery, PLGA forms nano capsules with superparamagnetic iron oxide nanoparticles (SPION) and cy7.5 for magnetic targeting, magnetic resonance, fluorescent molecular imaging, etc. (Grayston et al., 2022). The strategy based on nanomedicine is a good candidate to synergize the advantages of intravascular administration. It could track hematopoietic stem cells through imaging tags, reduce drug doses with a sustained release, or improve brain targeting distribution through functional materials (Grayston et al., 2022). Although nanoparticles can prolong the half-life of drugs and reduce systemic toxicity, their clearance accumulation and kinetics still need to be studied.

PEG is extensively used for nano-carrier surface modification, which is characterized by good hydrophilic and biocompatibility. PEG is used to modify the surface of pH-responsive rapamycin (RAPA) loaded treatment system, overcoming the difficulties of poor solubility, rapid metabolism and large side effects of the drug itself (Cheng et al., 2021). The results showed that mPEG-b-P (DPA-co-HEMA)-Ce6/RAPA NPs had good biocompatibility, efficient RAPA loading and acid enhanced dual-mode imaging ability (Cheng et al., 2021). In the transient middle cerebral artery occlusion (MCAO) rat model, the nano delivery system successfully monitored the drug distribution and located the cerebral ischemic area through magnetic resonance imaging (MRI) and near-infrared fluorescence (NIRF) imaging. Similarly, PEG-urokinase (PEG-UK) nanoparticles provided a dual targeting delivery of UK to both the large vessels and the microcirculation (Nan et al., 2021). In MCAO rat model, PEG-UK significantly reduced neurological score and infarct volume, and reduced the risk of bleeding (Nan et al., 2021).

PEG also formed block copolymers with a variety of polymers, which were used as drug carriers for stroke treatment. Guo et al. used PEG, poly ε-caprolactone (PCL), and peptide-digested block copolymers to construct protease-responsive brain-targeted nanoparticles (ASNPs), respond to proteases enriched in the ischemic microenvironment, including thrombin or matrix metalloproteinase-9 (Guo et al., 2018). ASNPs improved the efficiency crossing the BBB, and significantly enhanced glibenclamide’s efficacy (Guo et al., 2018). Hisayuki et al. designed and developed an NO radical having the tendency to self-assemble and form nanoparticles (NO RNPs) (Hosoo et al., 2017). RNP treatment retained the tight junction of ischemic brain tissue, inhibited neuronal apoptosis and oxidative stress injury, and increased the scavenging capacity of ROS to OH, ROO and O_2_ (Hosoo et al., 2017). The results showed that after cerebral ischemia-reperfusion injury, intra-arterial injection of RNP could reduce the damage of BBB and infarct volume by increasing the scavenging capacity of various ROS. Similarly, PEG-b-PMNT block copolymers, polyacrylic acid and tPA have been reported to form self-assembled cationic polymer nanoparticles (t-PA@iRNP) containing nitroxide radical (Mei et al., 2019). T-PA@iRNP pH-dependently released the encapsulated drug at the ischemic site, and significantly reduced ipsilateral subarachnoid hemorrhage area under thrombolysis coordination and antioxidation (Mei et al., 2019). It could not only prevent the rapid systemic clearance and enzymatic degradation of tPA, but also regulate the oxidative stress during reperfusion.

Polyamine dendrimers (PAMAM) are efficient gene carriers with anti-inflammatory properties. Lee et al. synthesized a second-generation dendritic macromolecule of PAMAM that coupled histidine and arginine to the primary amine of PG2 to form PG2HR, improving gene delivery efficiency and reducing cytotoxicity (Lee et al., 2021). PG2HR condensed the plasmid DNA into nanoscale complex by electrostatic action, and passed through the BBB by endocytosis of grid-independent protein. It improved the transfection efficiency and therapeutic outcomes. The heme oxygenase-1 (HO-1) gene, which has anti-inflammatory and anti-apoptotic effects, was used to assess performance of PG2HR. Compared with PO-1/PEI25K, PO-1/PG2 and PO-1/PG2R complexes, PO-1/PG2HR complexes decreased the level of apoptosis and infarct size. Thus, PG2HR had low cytotoxicity and high gene transfection efficiency (Lee et al., 2021).

Polyethylenimine (PEI), a cationic polymer, is extensively used for gene transfection. Hypoxia-specific anti-RAGA peptide (HSAP), heme oxygenase-1 (HO-1) plasmids and deoxycholic acid-conjugated polyimine form self-assembled nanoparticles through charge interaction for the treatment of ischemic stroke (Oh et al., 2019). RAGA is overexpressed in the ischemic brain and induces inflammation, and thus HSAP not only mediates efficient entry of cells into ischemic tissues through specific receptors, but also can inhibit RAGE-mediated signaling pathways to reduce inflammation (Oh et al., 2019). It could reduce the inflammatory reaction and tissue damage of ischemic brain tissue. HSAP NP overcame the nonspecific transmission of therapeutic genes and reduced the occurrence of side effects such as tumor growth, which was caused by nonspecific delivery of therapeutic genes.

Poly-n-isopropylacrylamide (PNIPAM) is a common thermos-responsive polymer, which is formed by free radical polymerization of n-isopropylacrylamide (NIPAM) with vinylpyrrolidone (VP) and acrylic acid (AA) (Haq et al., 2017). PNIPAM has been widely used in the preparation of thermal-response systems for biomedical applications. Curcumin is a plant polyphenol extracted from the rhizome of Curcuma in the family Zingiberaceae. It has anti-oxidative stress, anti-inflammation, anti-fibrosis and other pharmacological effects, and is extensively used in the prevention and treatment of cerebrovascular diseases. However, its poor bioavailability, low solubility, instability in body fluids and fast metabolism limit its therapeutic application. Ahmad et al. prepared PNIPAM nanoparticles (NPS) by free radical polymerization, which were loaded with curcumin (CUR), demethoxycurcumin (DMC) and double demethoxycurcumin (BDMC) (Ahmad et al., 2016). After intravenous injection, the neurobehavioral activities (motor ability and grip strength) were improved, and the levels of cytokines (TNF-α and IL-1β) were decreased (Ahmad et al., 2016). In general, polymer carriers increase the stability of volatile drugs and can be modified by surface to increase targeting or biocompatibility. However, relatively high cost and complex preparation process restrict its large-scale production.

#### Natural polymers

Natural polymers and their derivatives have been widely used for nanocarriers benefiting from their excellent biocompatibility and biodegradability. Natural polymers have the advantages of delaying or controlling drug release, stabilizing and protecting drug active ingredients. They can also increase drug absorption and distribution, and improve bioavailability (George et al., 2019).

As an important natural polysaccharide, starch is safe and non-toxic, with good biocompatibility and biodegradability. Starch is a potential drug carrier material with wide source and low price (Tharanathan, 2005). Hyperbranched cationic amylopectin derivatives are combined with 3-(dimethylamine)-1-propylamine to form gene vectors (DMAPA-amyp) (Deng et al., 2019a). RGD polypeptides were then used to modify the surface of nanoparticles (RGD-DmapA-amyp/HIF-1α-AA) and bind to the highly expressed integrins at the ischemic site. RGD-DMAPA-AMYP/HIF-1α-AA crossed the BBB *via* receptor-mediated endocytosis. It had good biocompatibility and high cellular uptake, and was a safe, non-viral gene carrier that could be endocytosed by human cells (Deng et al., 2019a). Moreover, it could significantly promote the formation of cardiovascular system *in vivo*. It was reported that hydroxyethyl starch was preparation of an integrin ligand-coupled pH-responsive double-targeted nanoparticle, SAG@PHSRN-HES (Yang et al., 2021a). By utilizing the increased expression of integrin α_5_β_1_ within the blood vessels of ischemic tissue, SAG@PHSRN-HES achieved ligand-mediated cerebral ischemia distribution. Smoothing agonists (SAG) promote angiogenesis and neuroplasticity, achieving pH responsive release at ischemic sites. The radius of SAG@PHSRN-HES is 31.52 nm, suitable for drug delivery of the BBB. The results showed that the water-soluble hydroxyethyl starch delivery platform successfully realized the drug loaded PHSRN-HES targeted delivery. It not only promoted angiogenesis and enhanced neural plasticity, but also increased drug enrichment and reduced the severity of ischemic stroke (Yang et al., 2021a).

**FIGURE 2:**
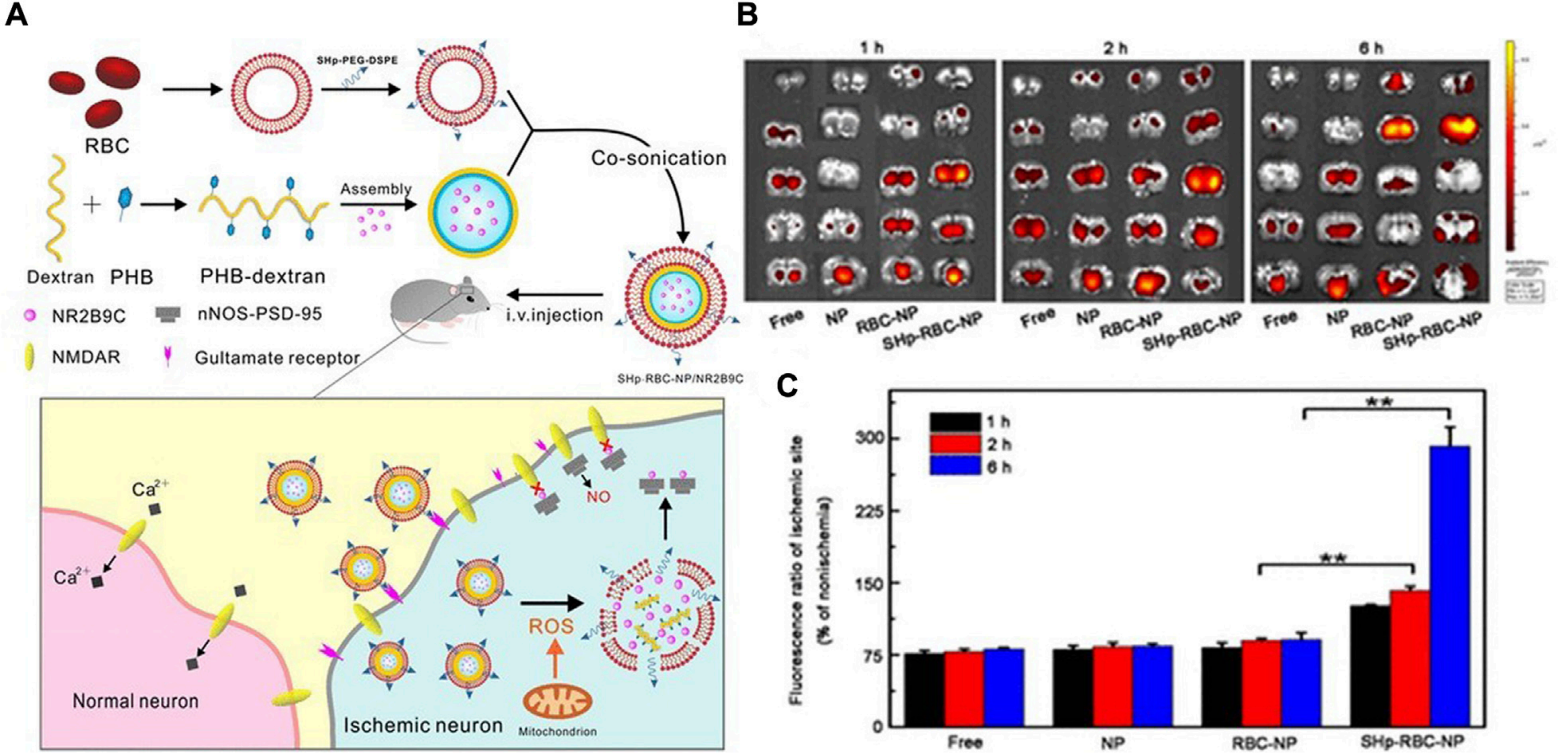
(A) Schematic design of the SHp-RBC-NP/NR2B9C. (B) Ex vivo fluorescent image of rhodamine-labeled free NR2B9C, NP, RBC-NP, and SHp-RBC-NP in the ischemic brain sections at 1, 2, and 6 h, respectively (n = 3). (C) Fluorescence ratio of ischemic hemisphere to the nonischemic hemisphere at 1, 2, and 6 h, respectively. Statistical analysis used one-way ANOVA test. It was permitted (Lv et al., 2018).

Dextran, the monomer of polysaccharide, has good biodegradability and biocompatibility (Esseku and Adeyeye, 2011). Diethylamine ethyl dextran (DEAE-dextran) is a kind of polyanionic reagent with high specificity. Based on DEAE-dextran, Jin et al. designed ROS-responsive 18β-glycyrrhetic acid-conjugated polymeric nanoparticles (DGA), which mediated neuroprotection of ischemic stroke through HMGB1 (high mobility group Box 1) (Jin et al., 2023). Microglia, an important innate immune component of CNS, is a double-edged sword in nerve damage because of its polarization between pro-inflammatory and anti-inflammatory properties. HMGB1 is one of the potent proinflammatory mediators that promote M1 polarization of microglia. Therefore, nanoparticles loaded with 18β-glycyrrhetinic acid (GA) could inhibit expression of HMGB1. DGA successfully improved low solubility and short biological half-life of GA, and significantly reduced the infarct volume (Jin et al., 2023). Similarly, dextran and erythrocyte membranes were synthesized into bioengineered borate modified dextran polymer nanoparticles (SHP-RBC-NP) with ROS-responsive behaviors. NR2B9C, a neuroprotective agent, could selectively disrupt the interaction of N-methyl-D-aspartate receptors (NMDARs) with the postsynaptic density protein (PSD-95) to prevent the overproduction of nitric oxide. As shown (Figure 2), when NR2B9C is internalized into ischemic neurons, NR2B9C is released from SHP-RBC-NPs due to high intracellular ROS levels and then selectively destroys NMDARs with PSD-95 to prevent nitric oxide overproduction. Because of the immune escape function of red blood cells, the circulation time of the two groups (RBC-NP and SHP-RBC-NP) was relatively long, showing excellent pharmacokinetic properties. This result was also demonstrated more visually by fluorescence imaging *in vitro*. The results showed that SHp-RBC-NP not only had a strong protective effect on glutamate-induced cytotoxicity of PC-12 cells, but also significantly prolonged the systemic circulation of NR2B9C in MCAO rats and enhanced the active targeting effect on ischemic regions (Lv et al., 2018).

Chitosan is a natural polysaccharide produced by chitin to remove some acetyl groups. It has many physiological functions, such as biodegradability, biocompatibility, non-toxicity, bacteriostasis, anti-cancer, lipid-lowering and immune enhancement. It is widely used for drug sustained release, gene transfer, medical absorbable and tissue engineering (Muxika et al., 2017). Gallic acid (GA) is a plant polyphenol that enhances the anti-inflammatory and antioxidant abilities, but it is less bioavailable and more metabolized (Fernandes and Salgado, 2016). GA-loaded o-hydroxymethyl chitosan nanoparticles (GA-NPs) could significantly increase the area under the plasma concentration-time curve and prolong the half-life of GA (Zhao et al., 2020). GA-NPs have a better therapeutic effect on ischemic stroke than GA, as reflected by the neurological dysfunction, cerebral infarction, inflammatory level and oxidative stress in the models of hypoxic glucose (OGD) and MCAO (Zhao et al., 2020).

Gelatin is a natural macromolecule protein with a structure similar to that of an organism (Djagny et al., 2001). Gelatin nanoparticles (GNPs) have been used as intranasal delivery carriers for osteopontin (OPN) in the treatment of ischemic stroke (Joachim et al., 2014). Intranasal administration was the process by which substances in the nasal cavity passed through the olfactory mucosa, following the olfactory nerve into the CNS (Gallardo-Toledo et al., 2020). It quickly and noninvasively delivers drugs to ischemic sites without crossing the BBB. GNPs reduced mean infarct size and extended the treatment for intranasal administration until at least 6 h after MCAO (Joachim et al., 2014). Gelatin could also be used to combine with polyhexadiol to form hydrophilic nanoparticles. Naringin is a potent anti-inflammatory that improves survival after brain transplants by reducing inflammatory stress in human mesenchymal stem cells. Ahmad et al. evaluated naringenin-loaded gelatin-coated polycaprolactone nanoparticles (nar-gel-c-PCL NPS), which successfully protected human mesenchymal stem cells from OGD-induced inflammatory responses by reducing levels of proinflammatory cytokine (TNF-α, IFN-γ, and IL-1β) and other inflammatory biomarkers (COX2, iNOS, and MPO activity) (Ahmad et al., 2019). Nar-gel-c-PCL NPs might be widely used in the treatment of ischemic stroke and other neuroinflammatory diseases.

Polysaccharide hydrogel is a cross-linked three-dimensional polymer network structure with large water absorption and high encapsulation efficiency (Bao et al., 2019). Fucoidan is a kind of natural algae-derived sulfated polysaccharide, which shows a strong tendency to over-expression of P-selectin in cardiovascular pathology (Fitton et al., 2019). Alina et al. prepared fucoidan functionalized hydrogel polysaccharide submicron (Fuco-SPs) with high biocompatibility by reverse microemulsion crosslinking method (Zenych et al., 2021). Due to the nanomolar interaction between fucoidan and P-selectin overexpressed in thrombotic activated platelets and endothelial cells, the gold standard fibrinolytic alteplase was used to guide site-specific fibrinolysis. It was found that Fuco-SPs showed a faster middle cerebral artery recanalization rate than free alteplase, and reduced cerebral infarction lesions after ischemia and BBB permeability (Zenych et al., 2021).

#### Inorganic nanomaterials

The different compositions of inorganic nanoparticles have unique physical and chemical properties, as well as a variety of forms and sizes, providing unprecedented opportunities for new biomedical applications. Inorganic materials play an important role in drug delivery and targeted therapy. So far, various inorganic nano antioxidants, such as carbon, cerium dioxide (CeO_2_), manganese dioxide and magnetite, have been designed to effectively treat stroke and other chronic diseases (Zhang et al., 2021a).

Nano sized iron oxide is a kind of multifunctional material with good optical properties, magnetism and catalytic properties. Biomimetic nanoparticles, composed of natural platelet (PLTs) membranes, L-arginine and γ-Fe_2_O_3_ magnetic nanoparticles (PAMNS), have been used for thrombus targeted transport of L-arginine and *in situ* formation of nitric oxide (NO) (Li et al., 2020). NO is an important signaling molecule that maintains vascular homeostasis, regulates vasodilation, and inhibits platelet activation and aggregation. Under the guidance of external magnetic field, PAMNS achieved the rapid targeting of ischemic stroke lesions. After releasing L-arginine at the thrombus site, endothelial cells produced NO, which promoted blood flow recovery and reperfusion of stroke micro vessels (Li et al., 2020). Magnetic nanocapsules (MNV) with mesenchymal stem cells derived from iron oxide nanoparticles (IONP) had good targeting properties (Kim et al., 2020). After systemic injection of MNV in MCAO rats, IONP could not only stimulate the expression of therapeutic growth factor in MSC, but also increase the localization of MNV to ischemic lesions by 5.1 times (Kim et al., 2020). MNV injection and subsequent magnetic navigation could promote the anti-inflammatory response, angiogenesis and anti-apoptosis of ischemic brain injury. Similarly, dm@LMNP, constituted with PLGA functionalized magnetic Fe_3_O_4_ nanoparticles (MNP), L-carnosine peptide (LMNP) and dexamethasone (dm), had the characteristics of controlled and sustained drug release. Dm@LMNP has been proved to be an effective drug delivery system for simultaneously crossing the BBB (Lu et al., 2021). In addition, iron oxide is used to construct the siRNA delivery system, siPHD2-EPCs-Alkyl-PEI/SPIO NPs, which can simultaneously realize siRNA delivery and nuclear magnetic resonance imaging (MRI) tracking endothelial progenitor cell (EPCs) (Wang et al., 2019). In the SIPHd2-EPCS group, infarct volume was significantly reduced and functional deficits and partial anisotropy (FA) values increased (Wang et al., 2019).

Cerium dioxide nanoparticles (CNPs) have high antioxidant activity and active oxygen scavenging capacity. This research showed that a new coating could enhance the biocompatibility of CNPs without weakening their antioxidant properties or increasing their toxicity (Kim et al., 2012). CNPs have strong hydrophobicity, and PEG modification can reduce nonspecific binding and organ uptake. He et al. proposed a strategy of *in-situ* synthesis of bioactive zeolites imidazole frame-8 coated cerium dioxide nanoparticles (CeO_2_@ZIF-8 NPs) to enhance catalytic and antioxidant activities and improve stroke treatment efficacy (He et al., 2020). CeO_2_@ZIF-8 NPs were characteristic with prolonging blood circulation time, reducing clearance rate and enhancing brain accumulation. It could effectively reduce lipid peroxidation, oxidative damage and neuronal apoptosis in brain tissue. CeO_2_@ZIF-8 can also inhibit astrocyte activation and proinflammatory cytokine secretion to reduce inflammatory and immune response-induced brain injury (He et al., 2020). Based on monodisperse ceria nanoparticles, Bao et al. prepared an effective stroke therapeutic agent (E-A/P-CeO_2_) with surface modification of angiopep-2 and PEG (Bao et al., 2018). Edaravone could synergistically remove ROS and significantly improved the removal efficiency of ROS. ANG improved drug distribution by binding to DLRP overexpressed on BBB cells. Therefore, E-A/P-CeO_2_ could effectively protect the BBB during treatment and greatly reduce adverse side effects and sequelae (Bao et al., 2018).

**FIGURE 3:**
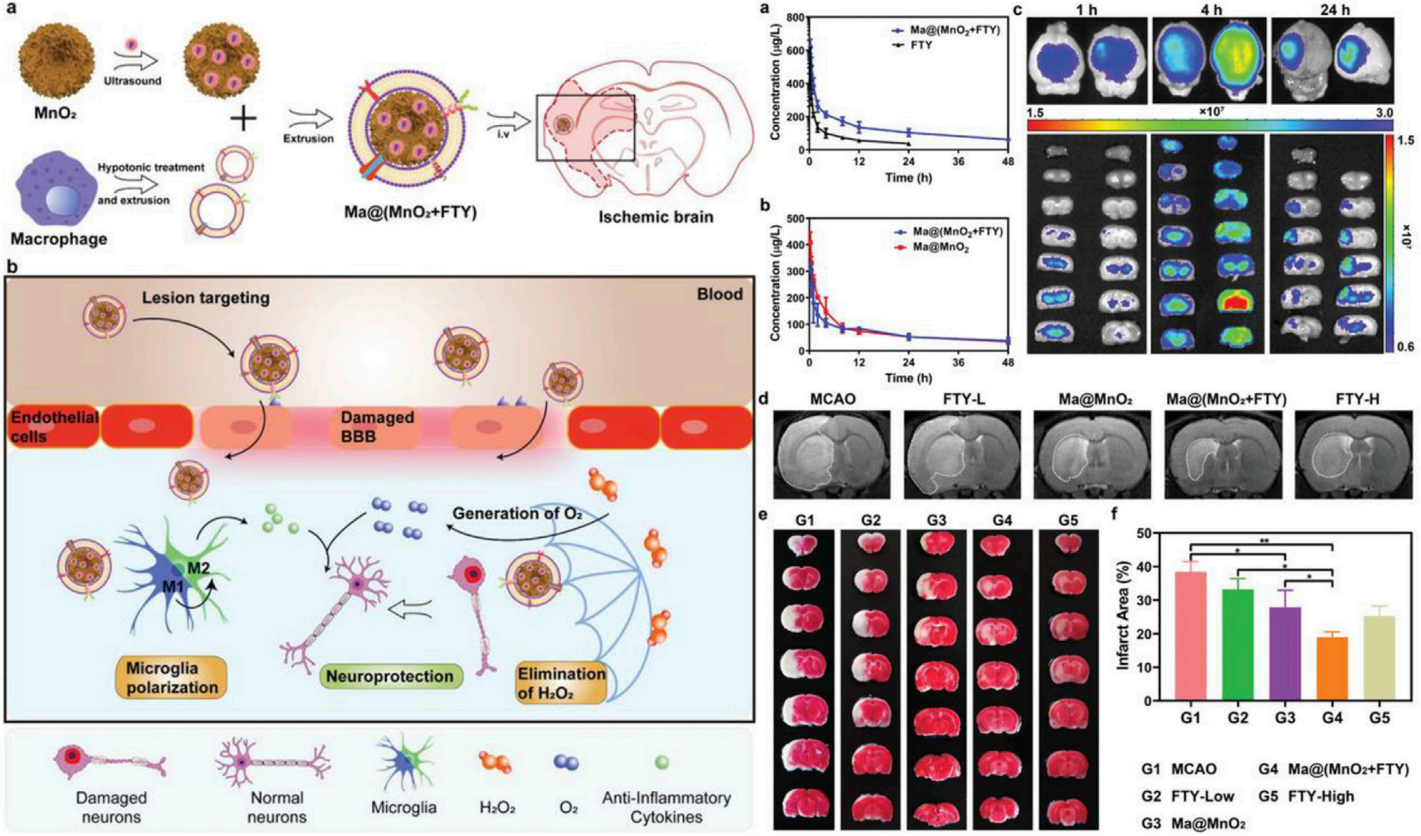
Left: Illustration of Ma@(MnO2 +FTY) nanoparticles formation and salvation of damaged neurons in ischemic brain. a) Scheme of the prepa-ration process of Ma@(MnO2 +FTY) nanoparticles. b) Illustration of Ma@(MnO2 +FTY) therapy for the rescue of ischemic penumbra. Coating with macrophage cell membrane increased the accumulation of Ma@(MnO2 +FTY) nanoparticles in the damaged brain. Ma@(MnO2 +FTY) could protect damaged neurons by consuming ROS, generating O2 and regulating proinflammatory microenvironment through promoting the phenotypic transition of microglia. Right: The pharmacokinetic behavior, targeting capability, and the rapeutic effect of as-prepared nanomedicine in vivo. a) The pharmacokinetic profiles of FTY after intravenous injection of free FTY and Ma@(MnO2 +FTY) nanoparticles with an equal FTY dose of 1.5 mg kg−1 . Data are presented as means ± SD, n = 3. b) The pharmacokinetic profiles of MnO 2 after intravenous injection of Ma@MnO2 and Ma@(MnO2 +FTY) nanoparticles at an equal MnO2 dose of 3 mg kg-1. Results are reported as means ± SD, n = 3. c) The targeting results of Ma@(MnO2 +FTY) nanoparticles to the ischemic brain, where fluorescence intensity in ischemic brain was observed at different times after injection of labeled nanoparticles. d) The infarct area of tMCAO/R rats treated with different drugs, monitored by MRI at 24 h post reperfusion. e) The rescue ability of nanoparticles on ischemic penumbra, brain sections were stained with TTC. f) The quantified results of TTC staining. Data are presented as means ± SD, n = 3, *P < 0.05, **P < 0.01.

Manganese dioxide nanoparticles, with strong antioxidant capacity, can significantly improve the anoxic conditions. It was reported that monodisperse hollow structured MnO_2_ (H-MnO_2_) could be obtained by *in situ* growth of MnO_2_ on solid silicon dioxide nanoparticles and removal of the silicon dioxide core (Li et al., 2021b). After modification of PEG, H-MnO_2_-PEG had better biocompatibility. H-MnO_2_-PEG effectively improved the cognitive ability after stroke, decreased ROS levels and increased survival. It also reduced cerebral infarct size, suppressed the production of inflammatory factors, and reduced apoptosis after stroke (Yang et al., 2021b). MnO_2_ particles camouflaged by macrophages could actively accumulate in the damaged brain through macrophage membrane protein-mediated recognition. FTY promoted microglial phenotypic transformation (M1 microglia to M2 type) through activation of signal transducers and transcriptional activator 3 (STAT3) pathways. Therefore, Ma@ (MnO_2_+FTY), made up of macrophage membrane, MnO_2_ and FTY, could reverse the proinflammatory microenvironment and enhance the survival of the damaged neurons (Li et al., 2021b) (Figure 3). In addition, Bovine serum albumin (BSA)-MnO_2_ nanoparticles (BM NPs) prepared by simulated disinfection were used for imaging BBB permeability in stroke patients (Hou et al., 2021). BM NPs had high *T*_*1*_ relaxation (*r*_1_ = 5.9 mM^−1^ s^−1^), remarkable imaging ability, and good biocompatibility, which could image the permeability of BBB with the advantages of non-invasive and timely (Hou et al., 2021).

All three inorganic particles described above have their own free radical scavenging properties, and iron oxide also has magnetic targeting capabilities. However, according to the current study, the vector itself may be potentially toxic and cannot be easily removed in the body, which may bring serious side effects.

### Endogenous carrier

Compare with above nanoparticles, membrane masking techniques have several advantages, including superior biocompatibility and the complex functions displayed by membrane donor cells under physiological or pathological conditions (Lv et al., 2018). Human endogenous cell-derived biomimetic drug carriers have higher biosafety and targeting capabilities than artificial carriers, providing new options for stroke therapy.

**FIGURE 4:**
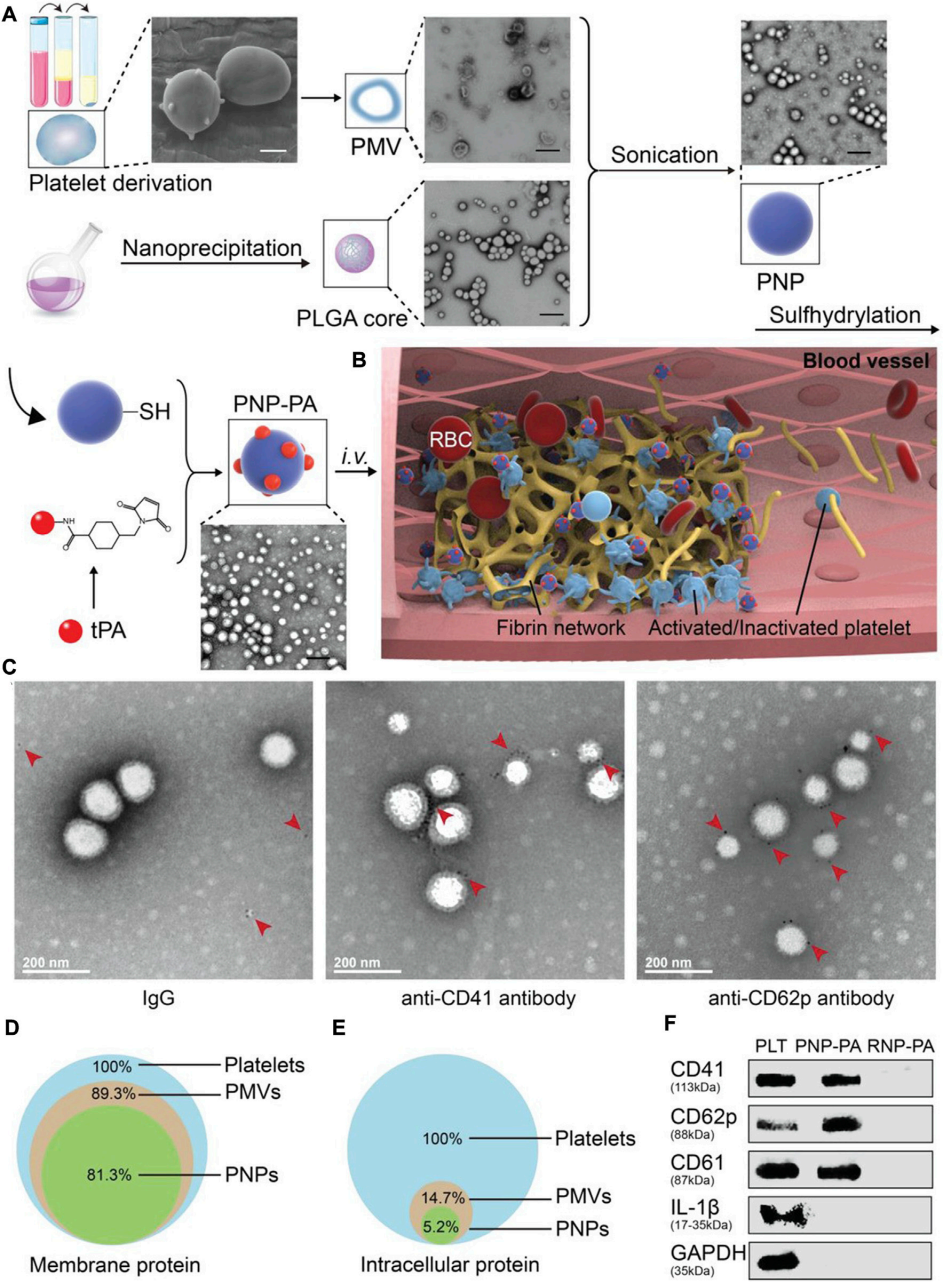
Schematic illustration of PNP-PA design and its characterization. a) Schematic illustration of the synthesis of PNP-PA nanoparticles. Briefly, the membrane of platelets (scale bar, 1 μm), acquired from the whole blood of mice, were used to coat the outside of the PLGA cores (scale bar, 400 nm). rt-PA was subsequently conjugated via –SH groups onto the surface of the platelet membrane to form PNP‐PA (PMV, PMVs; scale bar, 400 nm). b) The proposed mechanism of action of PNP-PA in thrombolysis. PNP‐PA specifically targeted to the thrombus site selectively dissolves the fibrin clot. c) Immunogold staining of CD41 and CD62p on PNP‐PA. Briefly, 10 μL PNP‐PA (1 mg mL‐1 ) solution was dropped onto ultrathin copper grids, incubated for 0.5 h at room temperature and then removed. The copper grids were next washed with PBS containing 1% BSA and 50 × 10-9 m glycine. 10 μL IgG, anti-CD41 or anti-CD62p antibody (0.5 mg mL‐1) were next dropped onto the copper and incubated for 30 min at room temperature and then blocked with 1% BSA for 15 min. The samples were incubated with 10 μL immunogold conjugate anti-Fc antibody (10 nm) containing 1% BSA for another hour. Finally, the samples were fixed with 1% glutaraldehyde for 5 min and stained with 10 μL 1% uranyl acetate for another 5 min and observed by TEM. d) Preservation of the platelet membrane proteins and e) intracellular proteins detected in PNP-PA and PMV. f) Western blot analysis of CD41, CD62p, CD61, IL‐1β, and GAPDH in platelets, PNP-PA and RNP-PA.

Platelet membrane camouflage nanocarriers may be able to prolong the circulating half-life of the drug and effectively target the site of platelet-rich thrombus. Platelet membranes extracted from mouse whole blood were used for PLGA surface binding to build nanoparticles (PNP-PA) (Xu et al., 2020). Rt-PA was coupled on the surface of PNP to achieve thrombus-targeted thrombolytic therapy. When administered intravenously in several different animal models of thrombosis, including mesenteric artery embolism and ischemic stroke, PNP-PA exhibits robust innate targeting and local clot degradation. These results suggested that the vector has therapeutic potential in the treatment of thrombosis-related diseases (Xu et al., 2020) (Figure 4). Similarly, PTNPs, consisting of paclitaxel and SPIO-stained PLGA nanoparticles as the inner core and platelet membranes as the coating shell, could be used to directly identify, intervene and monitor inflammatory neutrophil (Tang et al., 2019). The results showed that PTNPs could release network intervention drugs by internalizing the specific affinity between platelets and polarized network elements, thus effectively reducing the network infiltration in ischemic regions. (Tang et al., 2019). Inspired by the important role of platelets in thrombosis, Xu et al. developed a bioengineering nano platelet (tP-NP-rtPA/ZL006e) for sequential site-specific delivery of recombinant tissue plasminogen activator (rtPA) and neuroprotective agent (ZL006e) (Xu et al., 2019). TP-NP-rtPA/ZL006E first restored cerebral blood supply through higher-performance rtPA, and then tP-NP-rtPA/ZL006e accumulate across the BBB in damaged nerve tissue. Finally, ZL006E was released from tP-NP-rtPA/ZL006e to protect the injured neurons in the ischemic penumbra. Platelet-based biomimetic vectors will provide new insights for the treatment and diagnosis of acute ischemic stroke, as well as potential applications in other inflammatory diseases.

One means of reversing brain injury after stroke is ischemia-reperfusion, which may cause inflammatory responses and secondary tissue damage. Adhesion of neutrophil cells to endothelial cells is the basis of ischemic stroke inflammation (Da Silva-Candal et al., 2017). Inspired by this interaction, Dong et al. reported a drug delivery system consisting of neutrophilic membrane-derived nanocapsules and Resolvin D2 (RVD2) (Dong et al., 2019). It could not only specifically target the inflammatory brain endothelium, but also enhance the remission of inflammation. The results showed that RVD2-loaded nanoparticles significantly reduced the inflammatory response to ischemic stroke and improved neurological function in MCAO model rats (Dong et al., 2019). Monocyte membranes have been developed as drug particles to alleviate inflammation. A functional nanoparticle (McM/RNPs) consisting of monocyte membranes (McM) and sirolimus nanoparticles was reported to reduce inflammation by blocking monocyte infiltration and inhibiting microglia proliferation (Wang et al., 2021b). The results showed that McM/RNPS can actively target injury sites and bind to inflammatory endothelial cells, greatly improving neural score and infarct volume (Wang et al., 2021b).

At present, there are many kinds of endogenous cell vectors. NETs consisted of erythrocyte membrane, TPA, biotin-streptavidin (DSPE-PEG-CHO) and indocyanine green (ICG). Combined with the high circulation characteristics of erythrocyte membrane, the thrombolytic characteristics of TPA and the near infrared imaging characteristics of ICG, NETs showed well in the treatment of ischemic stroke and thrombosis imaging (Vankayala et al., 2018). Stem cell-derived extracellular vesicles had also become novel therapeutic effectors for immune regulation. But stem cells themselves were poorly targeted, hindering their further development. Tian et al. generated a recombinant fusion protein (RGD-C1C2) containing arginine-glycine aspartic acid (RGD)-4C peptide (ACDCRGDCFC) and the phosphatidylserine (PS) binding domain (C1C2). RGD-C1C2 binds to the EV membrane and was used to form RGD-EV_ReN_ for targeted treatment of ischemic brain tissue injury regions (Tian et al., 2021b). After intravenous administration, RGD-EV targeted the ischemic brain injury region and had a strong inhibitory effect on inflammatory response. Endogenous carrier is one of the best carriers in the future. They are inherently targeted, biocompatible and less likely to be attacked by the immune system. In comparison, blood cells have a longer cycle life. Platelets have a high storage capacity, but may lead to the risk of excessive clotting, shortening the lifespan of platelets.

### Liposome

Liposome membrane is mainly composed of phospholipids and cholesterol. Phospholipids, as the basis of liposome membrane structure, can form a relatively stable closed vesicle structure with a bimolecular layer due to their amphiphilicity (Wang and Grainger, 2019). Intact liposomes can cross the BBB and enter the brain through phagocytosis by monocytes in the circulatory system, which increases the aggregation of drugs at the targeted sites and improves the drug efficacy. For example, liposome-encapsulated acetate administration extended the half-life of acetate in the blood and reduced gastrointestinal irritation (So et al., 2019).

**FIGURE 5:**
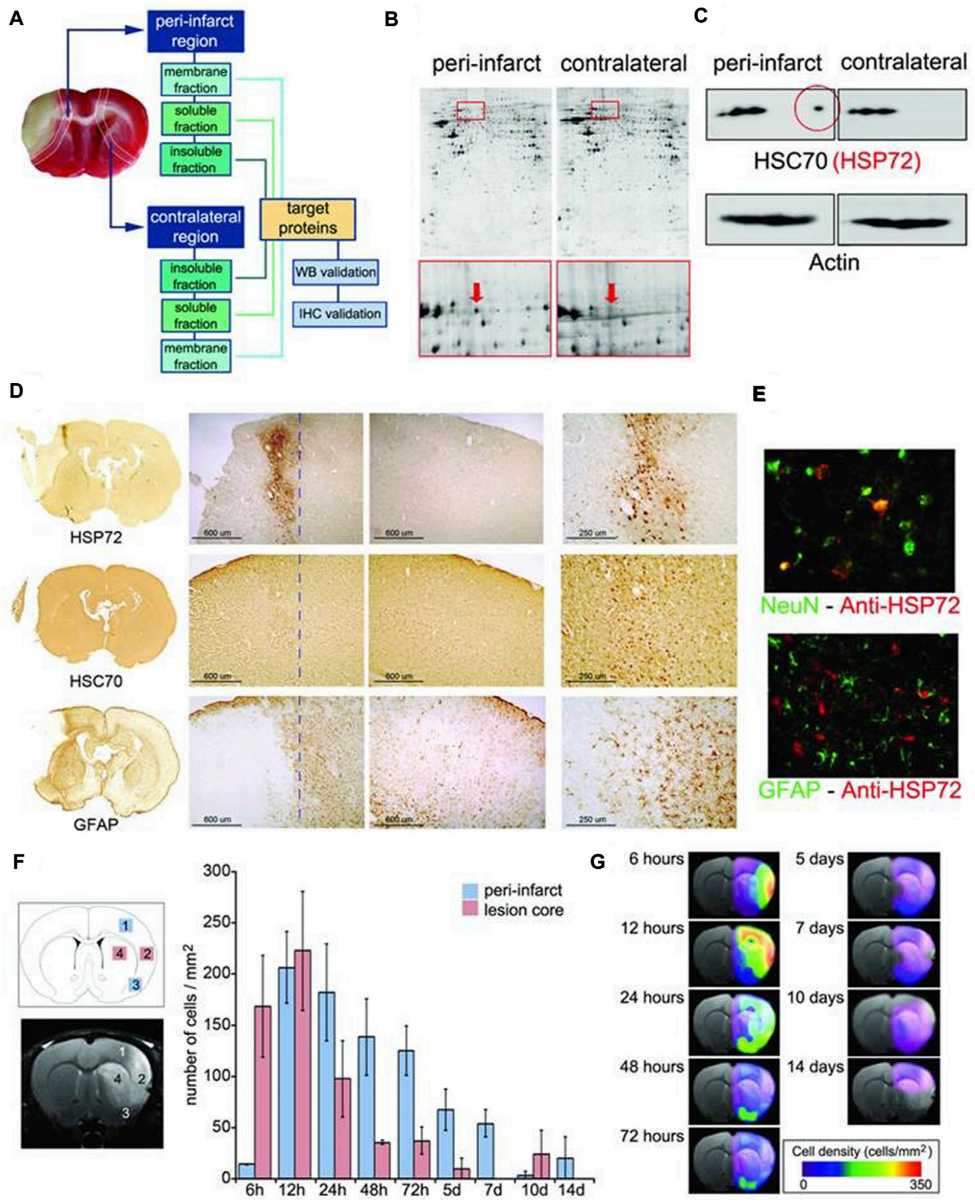
**(A)** Infarct core of a section of an ischemic rat brain stained (in beige) by tetrazole chloride. **(B)** Proteomic gels showing the expression of peri-infarct and contralateral proteins (spots) with magnification (red frames) of the region where HSP70 proteins is located. **(C)** 2D-WB results showing (top row) that HSP72 (circled in red) is the only member of the HSP70 family specifically expressed at the peri-infarct tissue, while other members of the family are also expressed in contralateral tissues (actin bands used for protein load control are also shown). **(D)** Immunohistochemical (IHC) study of consecutive brain slices (10 µm) stained for HSP72 (top-row), HSC70 (middle-row) and GFAP (bottom-row). **(E)** Double fluorescence IHC of peri-infarct tissue. HSP72 (red) is mostly expressed by neurons (NeuN green, top) but not by astrocytes (GFAP green, bottom), 48 h after ischemia. **(F)** Temporal profile of HSP72 expression in the ischemic brain. Regions selected for cell counting analysis are indicated in the MR image (where infarct core appears hyper-intense). **(G)** Color-coded density maps (overlaid on MR images) presenting the spatiotemporal expression profile of HSP72 in the ischemic brain. It was permitted (Agulla et al., 2013).

The pathological environment of ischemic stroke is very complex, so it is necessary to know the expression of each protein receptor before designing targeted nanoparticles. The peri-infarct tissue is a key target for the treatment of cerebral ischemia. Agulla et al. reported a new nano-therapeutic platform based on the peri-infarct tissue (Agulla et al., 2013). Expression of so-called molecular biomarkers in surrounding infarct tissue was examined. The results showed that HSP72 protein was a suitable biomarker for the periinfarct area, because it was selectively expressed by dangerous tissues within 7 days after cerebral ischemia. Finally, anti-HSP72 stealth immunoliposomes containing a large number of citicolines, fluorescent probes and imaging probes were used to delineate the peri-infarct area (diagnostic function) *in vivo* (Agulla et al., 2013) (Figure 5).

Wu et al. reported a novel preparation method for efficiently loading hydrophobic drugs (endogenous high hydrophobic molecular oil ethanolamide with significant neuroprotective effects) to liposome for stroke treatment (Wu et al., 2021). The effective retention of oleoylethanolamide (OEA) in liposome could significantly enhance neuroprotective effects. Liposome could significantly improve the survival rate, behavior score, cerebral infarction volume, edema degree and spatial learning and memory ability of MCAO model rats (Wu et al., 2021). Resveratrol (NR), found in grapes and red wine, has good antioxidant and anti-inflammatory properties (Malaguarnera, 2019). NR after encapsulated in nanostructured lipid carriers (NLCs) could effectively ameliorate the progress of ischemic stroke with low dose (Ashafaq et al., 2021). Zhao et al. designed a novel neuroprotective agent (ZL006) using liposome as a carrier, combining T7 peptide (T7) and stroke homing peptide (SHp), to penetrate the BBB and target ischemic regions (Zhao et al., 2016). Compared with non-targeted liposomes, T7&SHp-P-LPs/ZL006 could significantly enhance the cellular uptake of PC-12 cells stimulated by excitatory amino acids and reduce apoptosis (Zhao et al., 2016). Prolonging the time window of thrombolytic therapy and improving the secondary I/R injury are the desirable methods for the treatment of ischemic stroke. Liposomal Fasudil (Fasudil-Lip) was used in combination with tPA to treat photochemically induced thrombus formation in MCAO model rats. The study found that liposomes accumulated in ischemic areas in a time-dependent manner after intravenous administration (Fukuta et al., 2017).

The synergistic treatment of stem cells and liposomes can improve anti-inflammatory effects and drug absorption. Sivelestat-loaded nanoparticles (NCLs) were used to treat oxygen-glucose treated dental pulp stem cells and mesenchymal stem cells, which mimic the environment of stem cells during ischemia-reperfusion (Prakash et al., 2021). NCLs protected the loss of cell membrane integrity and restored cell morphology. Furthermore, NCLs successfully defended human DPSCs and MSCs against OGD-induced oxidative and inflammatory stress (Prakash et al., 2021). NBP is a multi-target drug for the treatment of ischemic stroke. It has high water solubility, but low oral bioavailability. Liposomes containing the biosurfactant sodium cholate have high biocompatibility and clinical application potential as oral NBP delivery platform. NBP-loaded CA-liposomes’ size is 104.30 nm and their release is 88% in 12 h, exhibiting rapid and almost complete drug absorption. 9-aminoacridine (9-AA) is a new activator of NR4N1 (Zhang et al., 2021b). Pang and his team combined liposomes with 9-AA to significantly reduce infarct size, improve neurological deficits and promote long-term functional recovery *in vivo* (Wang et al., 2020). Liposomes are less toxic and can deliver hydrophilic and lipophilic compounds. But it will be rapidly metabolized in the body, and the long-term storage stability is poor.

### Other nanocarriers

There are other bioactive materials involved but not widely used for the treatment of ischemic stroke. Betulinic acid (BA) is a natural antioxidant with antiviral, anti-diabetes, hypolipidemic and anti-inflammatory activities (Saneja et al., 2018). After intravenous injection, it could effectively penetrate the brain as an antioxidant and significantly reduce ischemia-induced infarction. Betulone amine (BAM), chemically transformed from BA, was used to build nanoparticles that preferentially released drugs in acidic ischemic tissue (Zhang et al., 2022). AMD3100, a CXCR4 antagonist, was used as a target molecule. Administration of A-BAM NPs not only enhanced the efficacy of NA1 (neuroprotective peptide), but also makes Na1 therapy compatible with tPA infusion (Zhang et al., 2022). In addition, BA NPs could load glibenclamide and enhance the transmission of glibenclamide, resulting in a significantly higher therapeutic effect than glibenclamide or BA NPs (Deng et al., 2019b).

Ischemic stroke can lead to irreversible neuronal damage, so the particle-targeted transport therapy has attracted much attention. Wheat lectin (Li et al., 2019), melanin nanoparticles (Liu et al., 2017), gold nanoparticles (Savchenko et al., 2016) and carbon materials (Fernandes et al., 2018) have been developed and utilized, and have achieved good curative effects to a certain extent.

## Conclusion

Ischemic stroke involves a variety of pathological mechanisms and is limited by pathological conditions, so it is difficult to find effective treatment. BBB is one of the biggest obstacles during treatment, which limits the transport of drugs in the brain. Therefore, the drug carrier which can assist drug to cross the BBB and release the drug effectively in the focus has become one of the research focuses. Among them, endogenous vectors become one of the most valuable vectors in the future because of their special immune escape and targeting functions. The poor stability of liposomes, the potential toxicity of inorganic particles and the complex process of polymers may be the direction of future research. Although a large number of studies have proved the role and efficacy of various drug carriers, the clearance of carriers and potential side effects are still unknown. In addition, in view of the complexity of ischemic stroke disease, nano delivery systems should also take into account the comprehensive protection of the nervous system. Most important of all, further research is needed to provide more possibilities from preclinical to clinical application.
