# Corrigendum: Advances in the research of nano delivery systems in ischemic stroke

**DOI:** 10.3389/fbioe.2023.1175184

**Published:** 2023-03-10

**Authors:** Yi-Xuan Li, Hong-Bo Wang, Jian-Bo Jin, Chun-Lin Yang, Jing-Bo Hu, Jing Li

**Affiliations:** ^1^ Faculty of Materials Science and Chemical Engineering, Ningbo University, Ningbo, China; ^2^ Department of Pharmacy, Ningbo University Affiliated Yangming Hospital, Yuyao, China

**Keywords:** ischemic stroke, blood-brain barrier, nano delivery system, polymer, endogenous vesicles

Incorrect affiliation

In the published article, there was an error in **Affiliations** 1 and 2. Instead of:

1. Department of Pharmacy, Ningbo University Affiliated Yangming Hospital, Yuyao, China,

2. Faculty of Materials Science and Chemical Engineering, Ningbo University, Ningbo, China

It should be:

“1. Faculty of Materials Science and Chemical Engineering, Ningbo University, Ningbo, China,

2. Department of Pharmacy, Ningbo University Affiliated Yangming Hospital, Yuyao, China.”

The authors apologize for this error and state that this does not change the scientific conclusions of the article in any way. The original article has been updated.

